# The next stage in Biology Open's support for early-career researchers

**DOI:** 10.1242/bio.059725

**Published:** 2022-11-23

**Authors:** Steven Kelly, Sophie L. Johnson

**Affiliations:** ^1^ Department of Plant Sciences, University of Oxford, Oxford OX1 3RB, UK

The term ‘early-career researcher’ (ECR) is misleading. ECRs aren't simply researchers; they attempt to answer the most important scientific questions of our time, while also taking on multiple other roles, including teacher, mentor, author and reviewer. As well as this, ECRs are at the forefront of future positive changes in academic publishing. For example, one study found that 30% of ECRs have a positive attitude to open practices (Nicholas et al., 2019) and 73% of ECRs in the Max Planck Society reported an intention to publish Open Access within the next year (Toribio-Flórez et al., 2021). Grassroots initiatives and advocacy groups, such as the eLife early-career ambassadors, are composed of cohorts of ECRs that are driving change towards open science, and greater integrity and equity in scientific publishing. It is clear that ECRs play a substantial role in supporting and helping to develop the future of scientific journals, but how are journals supporting and helping to develop the careers of ECRs?

Multiple journals provide a platform for their ECR author communities. Alongside published articles, they also produce podcasts, webinars, training programs and community spaces that are focused on ECRs. Several journals also offer travel grants and awards that support ECR research. Moreover, the growing popularity of social media for academic purposes (Nicholas et al., 2020) has put pressure on ECRs to cultivate a visible online presence, which can be achieved through publishing and engagement with journal initiatives. Journals can therefore support the ECR community by providing a platform for ECR content, by highlighting their work and by providing career-development opportunities. It is also important to give ECRs a voice by openly recognising their contributions to peer review and including them on editorial boards. Here at Biology Open (BiO) and The Company of Biologists, we try to do all of this and more, because supporting ECRs is one of our key goals (Kelly, 2021) (Box 1).

**Box 1. How The Company of Biologists supports ECRs**

BiO is published by The Company of Biologists, a not-for-profit publishing organisation dedicated to supporting and inspiring the biological community. The Company uses its income for the benefit of biology and biologists. Many of its activities focus on supporting ECRs in the vital first stages of their academic careers, offering a number of practical ways to meet their unique needs and the challenges they face (see https://www.biologists.com/about-us/early-career/).

**Facilitating scientific collaboration**

Travelling Fellowships of up to £3000 are offered to graduate students and post-doctoral researchers wishing to make collaborative visits to other laboratories (https://www.biologists.com/travelling-fellowships/).

Conference Travel Grants (up to £600) from sister journal Disease Models & Mechanisms are aimed at ECRs wanting to attend scientific meetings, conferences, workshops and training courses relating to the areas of research covered by the journal.

**Communication and blogging**

ECRs might consider writing for one of our community sites – the Node and FocalPlane – or even joining our ever-expanding group of preLighters writing for preLights (the preprint highlighting service). These dedicated community sites provide scientists with a place to interact, learn and discuss science informally.

**Future Leader Reviews program**

BiO created the Future Leader Reviews program specifically to help ECRs establish an identity and demonstrate independence in their chosen field. They can also be used to help people build their profiles ahead of those all-important grant, fellowship and job applications.

**Hosted internships**

We offer professional internships for PhD students (funded through PhD programmes). Each internship is carefully defined, both with the institute and the individual, and usually involves projects with our journal teams. During 2021-2022, the BiO Editor-in-Chief hosted an intern focused on supporting BiO ECR initiatives.

**Meeting Reviews**

BiO publishes Meeting Reviews. These provide a platform to support the dissemination of and access to meeting content for the global biological sciences community. Any meeting or conference that has received funding support from The Company of Biologists is eligible to apply to publish a **free** Open Access Meeting Review in BiO, but BiO requires that at least one of the authors be an ECR, such as a PhD student or post-doctoral researcher.

**Funded Workshop places**

Our Workshops provide a stimulating, and equal, environment for leading experts and ECRs. There are usually 30 participants at each Workshop, including 10 places that we fund for ECRs to join. Everyone attending speaks for the same amount of time and ECRs gain one-to-one access to leaders in their field.

Adapted from Kelly, 2021.

To try to go the extra mile to help build the careers of ECRs, we created the Future Leader Reviews program (https://journals.biologists.com/bio/pages/reviews) specifically to help ECRs establish an identity and demonstrate independence in their field. Although our Future Leader Reviews program is going well, we acknowledge that it can be a daunting task for an ECR to take on an entire field. Specifically, it can be challenging to critique and analyse all relevant findings and to discuss future prospects, challenges and questions that are yet to be addressed. Thus, to create more opportunities for ECRs to engage with scientific publishing, we have launched the next phase of our ECR-led articles: the ‘A Year at the Forefront…’ series (https://journals.biologists.com/bio/pages/yatf). A Year at the Forefront... articles are distinct from Future Leader Reviews in that they aim to highlight the key discoveries, technological innovations, new resources and new hypotheses that have made an impact in a specific biological field **during the past year only**. Our hope is that these articles will bring coherence to rapidly progressing fields, with emphasis on emerging science and cutting-edge research. Importance is therefore placed on the contributions of preprints to scientific advancement. Like our Future Leader Reviews program, these articles are intended to be written by ECRs who have less than 10 years’ active research experience post-PhD (excluding career breaks), independent of career stage. Any ECR can apply to write an article by completing the proposal form. As BiO is an Open Access journal that exists to profit science, not shareholders, and as we are committed to supporting and inspiring our ECR authors, we publish all of our review-type articles for free. Therefore, you don't need to have funding to publish, or a subscription to read, an A Year at the Forefront… or Future Leader Review. We will also promote your article on our social media, and of course BiO is indexed in Pubmed, PMC, Scopus and Web of Science.

**Figure:**
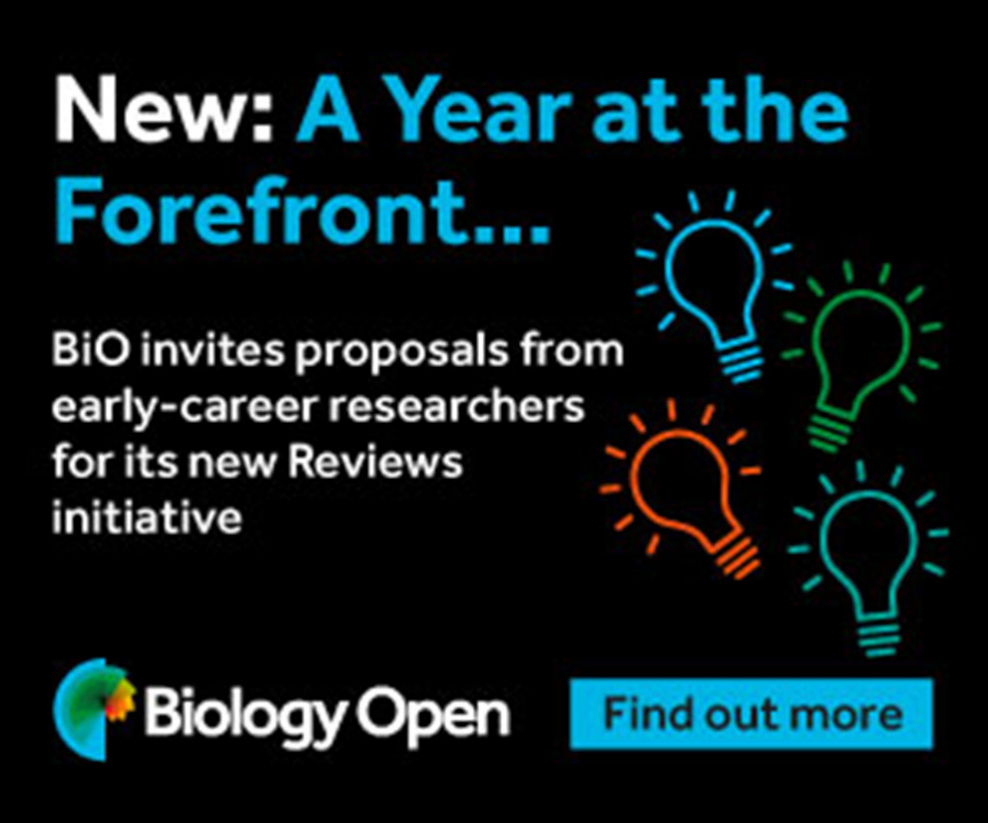


We hope that this new initiative will become a valuable addition to the scientific literature and to BiO's support of our ECR community. You can get an idea of what your A Year at the Forefront… article might look like by viewing our first published article, courtesy of BiO intern Sophie Johnson (Johnson, 2022). We hope it might inspire you to put pen to paper and look forward to receiving your proposals.
